# Successful Treatment of Unilateral Pulmonary Edema as Minimally Invasive Mitral Valve Surgery Complication—Case Presentation

**DOI:** 10.3390/jcm13247654

**Published:** 2024-12-16

**Authors:** Marius Mihai Harpa, Sânziana Flamind Oltean, Hussam Al Hussein, David Emanuel Anitei, Iulia Alexandra Puscas, Cosmin Marian Bănceu, Mihaly Veres, Diana Roxana Opriș, Radu Alexandru Balau, Horatiu Suciu

**Affiliations:** 1 Department of Surgery IV, George Emil Palade University of Medicine, Pharmacy, Science and Technology of Targu Mures, 38 Gheorghe Marinescu Street, 540139 Targu Mures, Romania; marius.harpa@umfst.ro (M.M.H.); alhussein.hussam@yahoo.com (H.A.H.); cosmin.banceu@umfst.ro (C.M.B.); v_misy@yahoo.com (M.V.); dianaroxana.opris@yahoo.com (D.R.O.); radu.balau@umfst.ro (R.A.B.); horisuciu@gmail.com (H.S.); 2 Regenerative Medicine Laboratory, Center for Advanced Medical and Pharmaceutical Research, George Emil Palade University of Medicine, Pharmacy, Science and Technology of Targu Mures, 38 Gheorghe Marinescu Street, 540139 Targu Mures, Romania; 3 Department of Cardiovascular Surgery, Emergency Institute for Cardiovascular Diseases and Transplantation Targu Mures, 50 Gheorghe Marinescu Street, 540136 Targu Mures, Romania; anitei_emanuel@yahoo.com (D.E.A.); alexandra.stoica92@yahoo.com (I.A.P.)

**Keywords:** unilateral pulmonary edema, minimally invasive mitral surgery, extracorporeal membrane oxygenator (ECMO), complications

## Abstract

**Background/Objectives:** In recent decades, the advantages of minimizing surgical trauma have led to the development of minimally invasive surgical procedures. While the benefits often outweigh the risks, several challenges are encountered that are not present in conventional surgical approaches. Unilateral pulmonary edema (UPE) after mitral interventions performed through a right-sided approach is a rare but potentially life-threatening event. **Methods**: We present the case of a 49-year-old patient who underwent endoscopic mitral valve repair. Immediately following ICU admission, the patient’s oxygen saturation suddenly dropped, and serous discharge was exteriorized from the endotracheal tube, with a thoracic X-ray revealing right-sided unilateral pulmonary edema. **Results**: The therapeutical course was complex. The patient developed hemodynamic instability, leading to cardiac arrest, which required cardiopulmonary resuscitation and the initiation of peripheral veno-arterial extracorporeal membrane oxygenation (VA-ECMO). The endotracheal cannula was replaced with a right-sided double-lumen cannula, and the patient was placed on two ventilators operating independently. The patient was weaned off extracorporeal membrane oxygenation (ECMO) on the fifth day and extubated on the sixth postoperative day. **Conclusions**: We successfully treated this patient using ECMO and independent lung ventilation. Several cases have been described in the literature, but the pathogenesis and risk factors of UPE remain unclear. Management depends on the severity of UPE, but a deeper understanding of its underlying mechanisms could provide cardiac surgeons with enhanced strategies for preventing UPE and implementing timely interventions.

## 1. Introduction

Unilateral pulmonary edema (UPE) has been reported after mitral interventions performed through a right-sided approach, representing a rare but potentially life-threatening event [1,2]. Minimally invasive cardiac surgery (MICS) has been shown to be highly successful, with low morbidity and mortality rates in high-volume centers. Although MICS offers significant benefits, such as reduced postoperative pain, improved respiratory function, less surgical trauma, shorter hospital stays, and enhanced aesthetic results, a critical issue with minimally invasive mitral valve surgery (MIMVS) is the occasional occurrence of unilateral pulmonary edema postoperatively [3,4]. Clinical signs manifest as respiratory or hemodynamic deterioration, and in severe cases, oxygenation impairment and cardiac failure may necessitate mechanical circulatory support [5]. The pathogenesis and risk factors of UPE remain incompletely understood. Various studies have investigated potential mechanisms, frequently identifying pulmonary re-expansion edema as the most likely cause. Other contributing factors include prolonged cardiopulmonary bypass (CPB) and aortic clamp durations, as well as inflammatory responses. Additionally, conditions associated with UPE following MICS include diabetes, chronic obstructive pulmonary disease (COPD), right ventricular dysfunction, pulmonary hypertension, and elevated perioperative C-reactive protein levels [6,7].

## 2. Case Report

This study presents a case of unilateral pulmonary edema occurring after MIMVS in a patient with pre-existing pulmonary disease that was effectively treated with veno-arterial extracorporeal mechanical support.

### 2.1. Patient Information

A 49-year-old man with a history of progressively worsening dyspnea and fatigue, diagnosed with severe mitral regurgitation, was referred to our clinic. His medical history includes stage II arterial hypertension, moderate pulmonary hypertension, asthma, dyslipidemia, and a COVID-19 infection one year prior to admission, complicated with pneumonia and parapneumonic pleural effusion. Informed consent for surgical intervention and publication was obtained from the patient, prior to surgery.

### 2.2. Preoperative Findings

An electrocardiogram showed sinus rhythm, with left ventricular hypertrophy. The laboratory findings were within normal ranges, except for a C-reactive protein (CPR) level of 0.95 mg/dL and lactate level of 1.62 mmol/L. A chest X-ray showed no significant pathological findings, and preoperative spirometry revealed moderate obstructive dysfunction, with a 75% FEV1.

With corticosteroid and long-acting bronchodilator therapy, the patient had no exacerbations of asthma, respiratory symptoms mainly being caused by the cardiac pathology.

Transthoracic echocardiography revealed severe mitral regurgitation due to posterior leaflet prolapse and flail from chordae rupture on a myxomatous mitral valve, moderate tricuspid regurgitation, tricuspid annulus measuring 36 mm, tricuspid annular plane systolic excursion (TAPSE) measuring 23 mm, and moderate pulmonary hypertension with a pulmonary artery pressure (PAP) of 55 mmHg. The left atrium measured 50 mm and the left ventricle 61 mm, with a preserved ventricular function, with an ejection fraction (EF) of 50–55%, and right ventricular function was also efficient. Cardiac catheterization showed normal coronary arteries. EUROSCORE II was 0.96%.

### 2.3. Intraoperative Data

**Figure 1:**
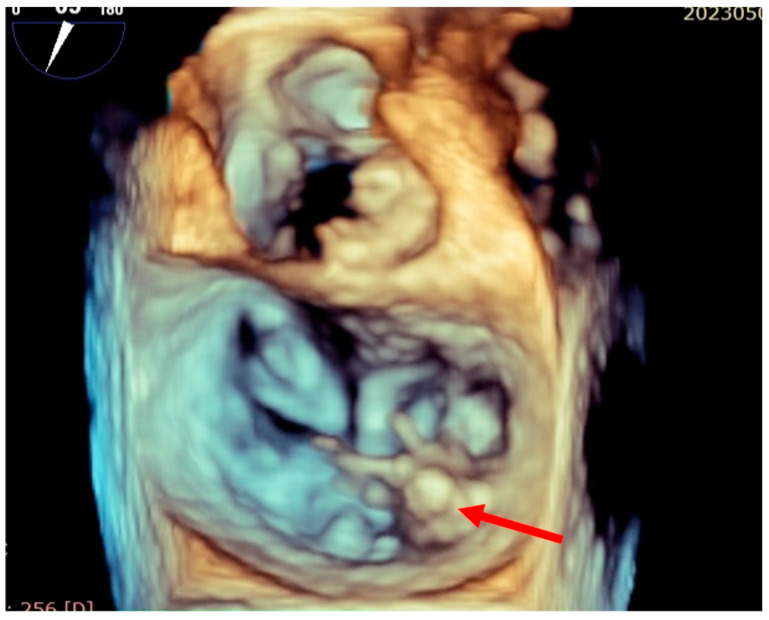
Intraoperative transesophageal echocardiography (3D reconstruction) revealing P3 flail due to chordae rupture (red arrow).

Intraoperative transesophageal echocardiography revealed a P3 flail due to chordae rupture and a P2-P3 cleft, with a mitral annulus measuring 45/39 mm (Figure 1). The patient underwent endoscopic mitral valve repair. We performed mitral valvuloplasty with four P2-P3 neo-chordae insertions and annuloplasty using a 36 mm Edwards Physio II ring through a right anterolateral mini-thoracotomy. Intubation was achieved with a single-lumen endotracheal tube, and ventilation was stopped after initiating CPB. Cardiopulmonary bypass was established via the right femoral artery and vein. The total CPB time was 185 min, with an aortic cross-clamping time of 118 min. Ventilation was resumed during weaning from CPB, and the procedure proceeded without complications. Intraoperative transesophageal echocardiography revealed trivial residual mitral regurgitation, an efficient left ventricle with an EF of 45–50%, and no impairment of right ventricular function. The patient was then transferred to an Intensive Care Unit (ICU).

### 2.4. Postoperative Evolution

**Figure 2:**
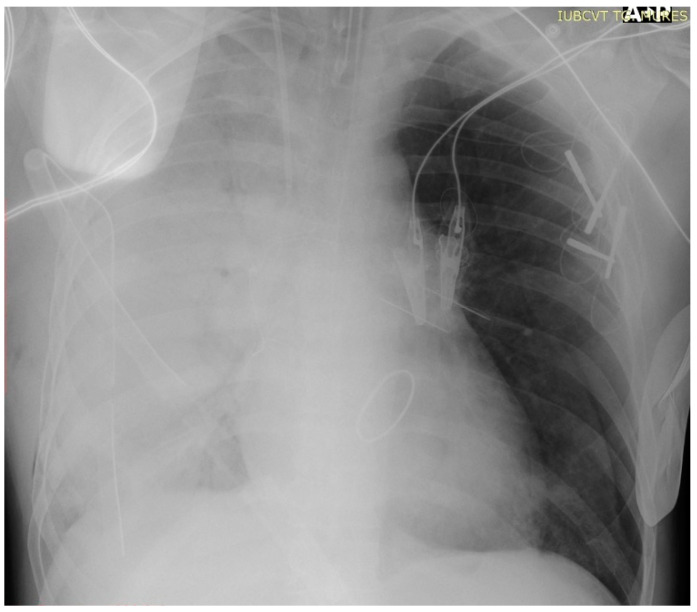
Thoracic X-ray showing right unilateral pulmonary edema.

Immediately following ICU admission, the patient experienced an abrupt onset of hypoxia, with oxygen saturation (SpO_2_) levels dropping to 70–75%. Arterial blood gas (ABG) analysis indicated an arterial partial pressure of oxygen (PaO_2_) between 40 and 50 mmHg. Serous discharge was aspirated through the endotracheal tube, and a thoracic X-ray revealed dense alveolar opacities throughout the right hemithorax, confirming unilateral pulmonary edema (Figure 2). During ECMO circuit preparation, the patient developed hemodynamic instability and cardiac arrest, requiring cardiopulmonary resuscitation. Right femuro-femural VA-ECMO support was initiated. Hemofiltration with a cytokine filter (CytoSorb, Monmouth Junction, NJ, USA) was also employed. Following the initiation of ECMO support, hemodynamic stability was restored, and oxygenation slightly improved. The vasoactive–inotropic score (VAS) increased from 61.9 before resuscitation to 215.4 during the process. However, a reduction in the required doses of vasoactive agents was observed a few hours after the establishment of mechanical support. Table 1 and Table 2 summarize the variations in arterial blood gas values and the inotrope and vasopressor requirements noted during resuscitation and after the initiation of ECMO support.

**Figure 3:**
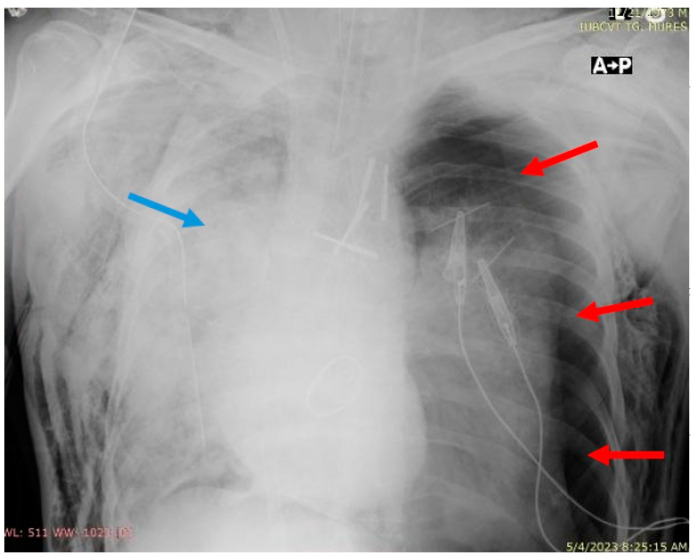
Thoracic X-ray revealing left pneumothorax (red arrows), UPE (blue arrow).

**Figure 4:**
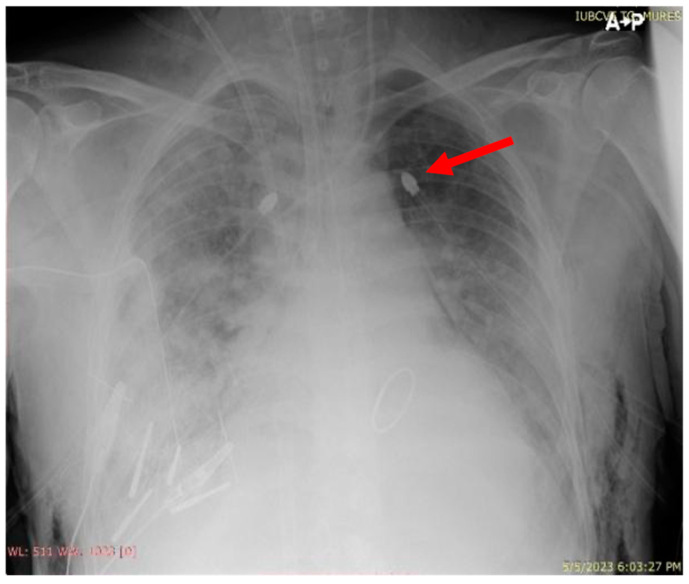
Thoracic X-ray after drainage (red arrow indicates drainage tube).

The day after surgery, the patient developed a left pneumothorax that required the insertion of a drainage tube (Figure 3 and Figure 4). Flexible bronchoscopy was performed and revealed mucus plugs, which required bronchoalveolar lavage and aspiration. The endotracheal cannula was replaced with a right-sided double-lumen cannula, and the patient was placed on two ventilators operating independently with the following settings: for the tracheal lumen, P-SIMV (Pressure Synchronized Intermittent Mandatory Ventilation), FiO_2_ 70%, P_n_sp. (inspiratory pressure) 13 cmH_2_O, PEEP (positive end-expiratory pressure) 3 cmH_2_O, with a respiratory rate of 16 breaths per minute, and for the bronchial lumen, P-SIMV, FiO_2_ 70%, P_n_sp. 20 cmH_2_O, PEEP 8 cmH_2_O, with a respiratory rate of 16 breaths per minute, which improved the patient’s oxygenation.

**Figure 5:**
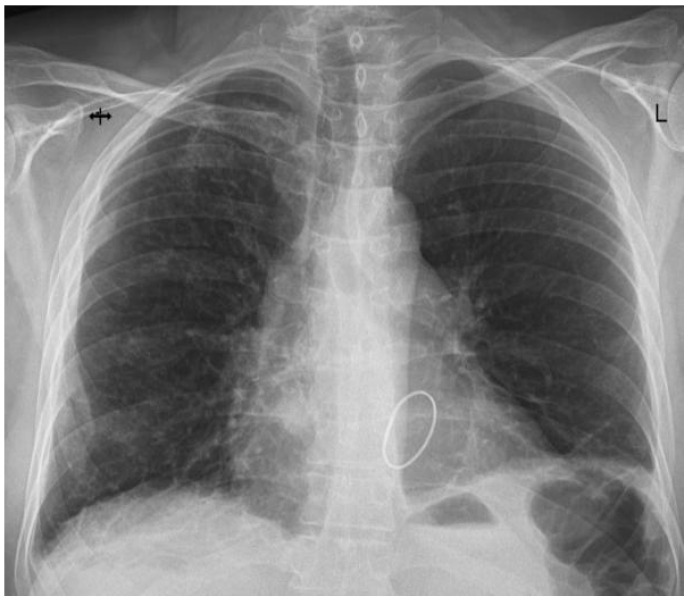
Thoracic X-ray at one month follow-up.

Ventilatory and ECMO parameters were adjusted based on the patient’s progress and clinical needs. The patient was weaned off ECMO on the fifth day and extubated on the sixth postoperative day. Throughout hospitalization, the inflammatory response was monitored through the daily measurements of C-reactive protein levels, which peaked at 19.24 mg/dL on the fourth postoperative day, followed by a progressive decrease to 2.15 mg/dL at discharge. Iatrogenic right pulmonary vein stenosis or occlusion was excluded via echocardiographic assessment and angio-CT scan. During the ECMO weaning process, severe right ventricular (RV) dysfunction was observed (TAPSE 7–11 mm), for which treatment with milrinone was continued. Additionally, a dose of Levosimendan was administered. The patient was monitored daily via echocardiography. A slight improvement in RV function was noted on the 10th day, with a TAPSE of 12–13 mm. The subsequent course was uneventful. A thoracic X-ray taken at the one-month follow-up is shown in Figure 5. At the one-year follow-up, the patient presented with no signs or symptoms of cardiac or respiratory failure and no pathological findings on echocardiography. The case timeline is succinctly outlined in Table 3.

Minimally invasive cardiac surgery has redefined the landscape of cardiac interventions by utilizing advanced surgical techniques, such as endoscopic and robotic assistance, through limited incisions often no larger than six centimeters. This approach allows surgeons to access the heart without the need for a full median sternotomy. Comparing classical approach with minimally invasive procedures, short-term and long-term mortality rates were similar, as were in-hospital morbidities, including renal, pulmonary, and cardiac complications, pain perception, and readmission rates. Additionally, there was a reduction in sternal complications, transfusion requirements, postoperative atrial fibrillation, duration of mechanical ventilation, and lengths of stay in both the ICU and hospital [8,9,10]. More than that, right mini-thoracotomy seems to be a safe alternative to traditional re-sternotomy for patients who have had previous cardiac surgery, in terms of hospital stay, ICU stay, and a lower risk of new-onset renal failure requiring dialysis [11].

However, minimally invasive cardiac surgery has potential limitations. A primary drawback is the increased technical complexity of the procedure compared to conventional open-heart surgery, necessitating highly experienced surgical teams and advanced instrumentation. Furthermore, patient selection is important, as MICS may be contraindicated in individuals with specific cardiac pathologies or anatomical considerations that preclude the safe or effective application of the technique. MIMS procedures are associated with prolonged operative times (cross-clamp time, CPB time) [12,13], with a steep learning curve [14], difficulties with de-airing and managing right ventricular dysfunction, a higher likelihood of reoperation for bleeding, and an increased risk of vascular complications [15]. A concern with the right chest approach is the reduced protection of the right ventricle (RV), as it cannot be directly cooled topically. However, this limitation can be addressed by employing lower systemic perfusate temperatures and administering cardioplegia more frequently to ensure adequate myocardial protection [12]. The use of retrograde perfusion via the femoral artery is associated with a higher incidence of neurological complications in older patients with atherosclerotic burden [16,17], although other studies showed a similar or lower incidence of stroke [12,18]. Complications associated with groin access, including lymphocele formation, vascular lesions, and infection, have become increasingly uncommon [19].

Single-lung ventilation (SLV) is often essential in MICS, particularly for lateral thoracotomy approaches, as it allows for an optimal visualization of the surgical field. However, SLV comes with its own challenges. The prolonged exclusion of one lung can result in ventilation–perfusion mismatch, barotrauma, and increased pulmonary vascular resistance. These effects may exacerbate hemodynamic instability and contribute to postoperative complications [20].

Unilateral pulmonary edema represents a rare but significant complication within the spectrum of MICS [21,22]. Several studies have reported on risk factors and have proposed preventive strategies; however, the mechanisms behind this pathology remain unclear [23,24]. While hemilateral atelectasis due to re-expansion is unlikely with single-lumen ventilation, microatelectatic areas can still form due to reduced ventilation in parts of the lung during the procedure. Upon re-expansion, localized inflammation and fluid leakage may occur, contributing to UPE. Localized trauma is another factor, as minimally invasive procedures often use specialized instruments and techniques that may inadvertently traumatize pulmonary capillaries or parenchymal tissues. Even heat from the video camera can be a local traumatic factor. Additionally, increased pulmonary venous pressure, due to the patient’s position, the inadequate drainage of pulmonary veins due to traction, or obstruction, can lead to stagnation and increased hydrostatic pressure, contributing to pulmonary edema [6,7,25]. Re-expansion pulmonary edema typically occurs after the re-expansion of collapsed lung regions (even after pneumothorax or pleural effusion drainage), which can involve increased capillary permeability and inflammatory responses. This mechanism has been documented even when the lung was not entirely collapsed but had reduced ventilation, as might occur during minimally invasive procedures [26,27]. Ischemia–reperfusion injury may also occur during the surgical procedure due to the temporary ischemia of the lung on the affected side from the manipulation of intrathoracic structures or alterations in regional blood flow. This phenomenon, characterized by oxidative stress, endothelial damage, and inflammatory responses, may be amplified by extended CPB durations. The severity of these pathological effects appears to correlate directly with the length of bypass time, underscoring the need for careful intraoperative management to minimize CPB exposure [28,29].

Prolonged CPB and surgical manipulation can trigger a generalized inflammatory response, as the interaction between blood components and the artificial surface of the CPB circuit stimulates the release of pro-inflammatory cytokines [30,31,32,33]. This response has significant clinical implications, with acute lung injury being one of the most critical complications, potentially leading to postoperative interstitial pulmonary edema and impaired gas exchange [34,35,36,37,38].

In our particular case, the development of this complication can be attributed to multiple pathophysiological mechanisms. Prolonged cardiopulmonary bypass is a well-recognized factor associated with adverse outcomes; however, its effects are not uniformly observed in all patients. In this instance, the patient’s pulmonary comorbidities—moderate obstructive dysfunction secondary to asthma, moderate pulmonary hypertension, and a history of severe COVID-19 infection—may have rendered the respiratory system particularly vulnerable to the disruption of homeostasis during surgery. The mitral valve was accessed via Sondergaard’s groove, a technique employing a self-retaining retractor and traction sutures to optimize visualization. While effective for exposure, this approach may transiently occlude or constrict the right pulmonary veins, leading to elevated pulmonary venous pressure. The resulting increase in hydrostatic pressure within the pulmonary vasculature is a recognized precursor to pulmonary edema, as noted in prior reports [6,39,40,41,42].

Despite the high mortality associated with UPE, there are no standardized treatment protocols [7]. Management depends on the severity of UPE: mild-to-moderate cases are treated with supportive care, including oxygen therapy and noninvasive or invasive ventilation, while ECMO is reserved for severe cases [43,44]. In cases of persistent respiratory failure despite ventilatory support, Veno-Venous (VV) ECMO is recommended. This strategy is indicated when there is severe hypoxia (SaO_2_ < 90% with FiO_2_ at 100%) and hypercapnia (PaCO_2_ > 45 mmHg) [45]. VV-ECMO facilitates extracorporeal gas exchange, allowing for lung-protective ventilation strategies [46]. However, in our case, VA-ECMO was used due to the patient’s hemodynamic instability. The onset of massive endotracheal secretions led to severe hypoxia, hemodynamic instability, and subsequent cardiac arrest. Resuscitation was performed prior to ECMO initiation, with peripheral VA-ECMO support quickly restoring hemodynamic stability. Based on our experience, ECMO should be promptly considered once severe UPE is identified or when signs of hemodynamic instability appear. Preventing the total collapse of the right lung can be achieved by maintaining positive end-expiratory pressure (PEEP) during CPB, adjusting to moderate-to-high PEEP and low tidal volumes to minimize the risk of RPE [3,47]. Independent Lung Ventilation (ILV) has been reported as a rescue strategy for patients with severe unilateral lung pathology. Using a double-lumen tube and two ventilators with different settings improves both oxygenation and hemodynamics [26,48,49]. In this case, we replaced the single-lumen endotracheal tube with a right-sided double-lumen tube and used two ventilators to optimize ventilation. The implementation of ILV was effective in managing this severe case of UPE. The intermittent ventilation of the right lung during surgery may reduce the risk of postoperative UPE. Additional protective measures include the administration of mannitol prior to aortic unclamping and maintaining mild hypothermia [23,50]. Approximately 2% of UPE cases in the immediate postoperative period may require ECMO as a rescue therapy [6,8,43]. Temporary VV-ECMO has been shown to support single-lung ventilation following CPB in lateral mini-thoracotomy for cardiac surgery, reducing the risk of postoperative UPE [51].

Patient selection seems to be essential for good results. The presence of morbid obesity, significant lung disease, peripheral vascular disease, advanced liver or renal disease, significant pulmonary hypertension, and severe left ventricle dysfunction are comorbidities of concern and can make patients less-than-ideal for MIMS procedures [19,52].

## 3. Conclusions

Several cases of unilateral pulmonary edema (UPE) following minimally invasive cardiac surgery (MICS) have been described in the literature, though its pathophysiology remains unclear. It is believed to involve a combination of systemic inflammatory response, direct mechanical injury, and ischemia–reperfusion effects. Although UPE is a rare complication, our aim is to raise awareness among MICS surgeons regarding its risk factors and underlying mechanisms. In our case, the prompt identification of UPE and the timely initiation of ECMO support led to a steady recovery of the patient. A deeper understanding of UPE’s underlying mechanisms could equip MICS surgeons with enhanced strategies for preventing UPE and implementing timely interventions. The immediate consideration of ECMO support is recommended upon the confirmation of severe UPE or if the patient exhibits signs of hemodynamic instability. Additionally, strategies such as differential lung ventilation, avoiding prolonged lung ischemia, and intermittent ventilation may help reduce the risk of postoperative UPE, although further studies are needed to confirm their effectiveness.

## Figures and Tables

**Table 1:** Arterial blood gases before, during, and after resuscitation and mechanical support.

Arterial Blood Gas Analysis (Right Radial Artery)						ECMO	
	Oxygen Saturation (%)	Partial Pressure of Oxygen (mmHg)	Partial Pressure of Carbon Dioxide (mmHg)	Ph (7.32–7.43)	Lactate (mmol/L)	O_2_ Flow (L/min)/FiO_2_ (%)	Flow (L/min)/RPM
1st hour PO	73	43	49	7.26	3.6	-	-
2nd hour PO (CA)	83	57	58	7.09	6.8	-	-
2nd hour PO (ECMO support)	99	76	57	7.20	7.7	4/100	4.7/3085
5th hour PO (cytokine filter)	97	89	51	7.27	12	4/100	4.4/2990
10 h PO	99	167	26	7.36	11.8	3.5/100	4.2/2810
12 h PO	99	174	28	7.37	10.9	3.5/100	4.2/2810
Day 1 PO	99	169	34	7.36	6.8	4.0/90	2.9/2395
Day 2 PO	100	170	54	7.21	4.5	4.5/60	2.4/2450
Day 5 PO	99	193	34	7.45	1.75	2.5/60	1.5/1831

PO—postoperatively; CA—cardiac arrest; ECMO—extracorporeal membrane oxygenation; RPM—rotations per minute.

**Table 2:** Inotropic score (IS) and vasoactive–inotrope score (VAS) before, during, and after resuscitation and mechanical support institution and following postoperative days.

	Noradrenaline (µg/kg/min)	Adrenaline (µg/kg/min)	Dobutamine (µg/kg/min)	Milrinone (µg/kg/min)	Vasopressin (UI/kg/min)	IS	VAS
Intraoperative following weaning from CPB	0.5	-	8.9	0.3	-	8.9	61.9
1st hour PO	0.7	0.2	11.1	0.3	0.002	31.1	124.1
2nd hour PO (CA)	1.4	0.2	11.1	0.2	0.003	31.1	203.1
2nd hour PO (ECMO support)	1.4	0.2	11.1	0.4	0.004	31.1	215.4
5th hour PO (cytokine filter)	1.4	0.2	11.1	0.4	0.004	31.1	215.4
10 h PO	1.4	0.2	8.9	0.2	0.004	28.9	210.9
12 h PO	0.7	0.1	8.9	0.2	0.004	18.9	130.9
Day 1 PO	0.6	0.1	8.9	0.4	0.0009	18.9	91.9
Day 2 PO	0.3	-	5.6	0.4	0.0007	5.6	46.6
Day 5 PO	-	-	1.5	0.2	-	1.5	3.5
Day 10 PO	-	-	-	0.3	-	-	3
Day 15 PO	-	-	-	0.1	-	-	1

CPB—cardiopulmonary bypass; PO—postoperatively; CA—cardiac arrest; ECMO—extracorporeal membrane oxygenation; IS = dopamine dose (µg/kg/min) + dobutamine dose (µg/kg/min) + 100 × epinephrine dose (µg/kg/min); VAS = IS + 10 × PDE inhibitor (milrinone) dose (µg/kg/min) + 100 × norepinephrine dose (µg/kg/min) + 10,000 × vasopressin dose (UI/kg/min).

**Table 3:** Case timeline.

Preoperative findings		The patient was referred for progressively worsening dyspnea and fatigue and diagnosed with severe mitral regurgitation. Transthoracic echocardiography revealed severe mitral regurgitations caused by posterior leaflet prolapse and flail due to chordae rupture.
Surgical intervention	Day 0	Endoscopic mitral valvuloplasty was performed, with four P2–P3 neo-chordae insertions and annuloplasty using a 36 mm Edwards Physio II ring, through right anterolateral mini-thoracotomy.
Postoperative evolution		Right after Intensive Care Unit (ICU) admission, hypoxia abruptly developed, and serous discharge was aspirated through the endotracheal tube. The thoracic X-ray performed revealed dense alveolar opacities on the entire right hemithorax, confirming the diagnosis of unilateral pulmonary edema.
		The patient developed hemodynamic instability requiring cardiopulmonary resuscitation. Peripheral VA-ECMO support was instituted during resuscitation, with the cannulation of the right femoral artery and vein, improving hemodynamic and respiratory parameters.
	Day 1	The patient developed a left pneumothorax, confirmed by thoracic X-ray, worsening respiratory parameters, therefore requiring the insertion of a drainage tube. A thoracic X-ray after drainage confirmed the correct positioning of the tube and the complete expansion of the left lung.
		A flexible bronchoscopy was performed and revealed mucus plugs that required bronchoalveolar lavage and aspiration.
	Day 3	The endotracheal cannula was changed to a double-lumen right-sided cannula, and the patient was placed on two ventilators operating independently. The parameters were adjusted based on the patient’s progress and needs.
	Day 5	The patient was weaned off from ECMO support. RV disfunction required prolonged milrinone and Levosimendan administration.
	Day 6	The patient was extubated.

## Data Availability

All the data can be found in the archive of Emergency Institute for Cardiovascular Diseases and Transplantation Targu Mures, Romania.
